# Lymphoid-like fibroblast reticular cells in the secondary lymphoid tissues and tumor microenvironment induce immunosuppression via maintaining the tolerogenic function of myeloid populations

**DOI:** 10.1186/2051-1426-2-S3-P201

**Published:** 2014-11-06

**Authors:** Yan Cui, Gang Guo

**Affiliations:** 1 Department of Biochemistry and Molecular Biology, Georgia Regents University, Augusta, GA, USA

## 

Recently, compelling evidence reveals that stromal populations, especially the fibroblastic reticular cells (FRC), of the secondary lymphoid organs (SLO) not only serve as structure scaffold for organizing and maintaining the structure of the lymphoid tissues, but also possess crucial functions in immune regulation, including induction of T cell tolerance and the generation of regulator T cells. Interestingly, we and others recently reported that similar lymphoid-like fibroblastic reticular cells also exist within the tumor microenvironment (TME) that facilitates the organization of tumors, as well as the induction of immune tolerance. We showed that those stromal cells augmented the differentiation of myeloid cells, including myeloid derived suppressors (MDSCs). As chronic inflammation plays a vital role in tumor initiation, progression, and metastases, we hypothesized that the crucial functions of these lymphoid-like FRCs in the TME are to induce immunotolerance and promote chronic inflammation, thereby promoting tumor progression. In this study, we examined the cellular and molecular characteristics of these FRCs and revealed their heterogeneity both at cellular and molecular levels. Interestingly, some of their properties appear to be intrinsic, not easily altered by the milieu they reside. Functionally, these FRCs provide crucial signals for the survival, differentiation, and tolerogenic phenotype of myeloid cells, including MDSC, dendritic cells, and macrophages. We are currently in the process of elucidating the molecular mechanism by which these FRCs induce tolerogenic myeloid populations. Our better understanding of the immune modulatory function and specific mechanisms of these FRCs, both within the lymphoid tissues and TME, will provide valuable information for the development of novel immunotherapy regimens to improve therapeutic outcome by combining active immunotherapy and the ultimate reversal of tumor-induced immunotolerance.

Supported by 01CA169133 from NCI/NIH and funds from GRU Cancer Center to YC.

